# Supplementary material for: The Evolution of Glycoside Hydrolase Family 1 in Insects Related to Their Adaptation to Plant Utilization
Source: Insects. 2022 Aug 30;13(9):786. doi: 10.3390/insects13090786 (PMC9500737; doi:10.3390/insects13090786)
Supplement: Supplementary file 1 [file insects-13-00786-s001.zip › Supplementary File S1.pdf]

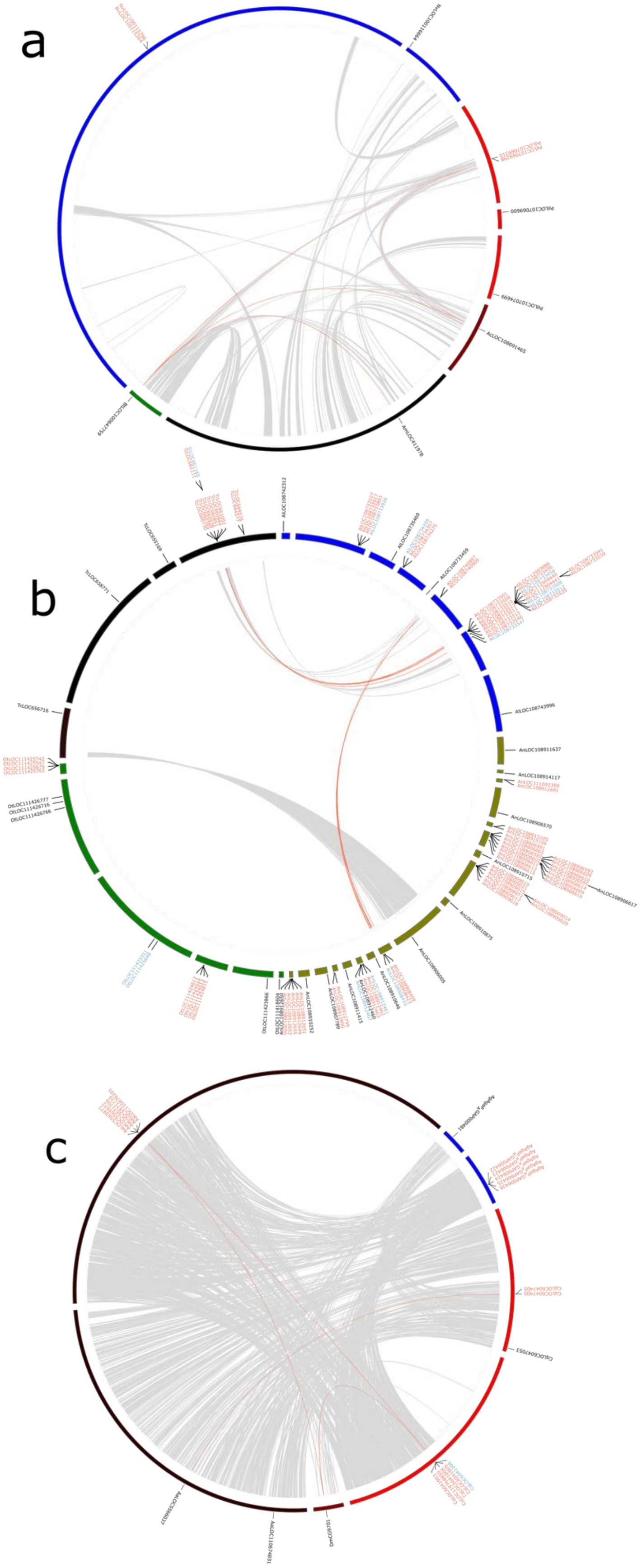
a
PdLOC107066600
PdLOC107074699
AcLOC108691465
AmLOC411978
b
AtLOC108742312
AtLOC108743996
TcLOC656716
AnLOC108911637
AnLOC108914117
AnLOC108906570
AnLOC108910715
AnLOC108906617
AnLOC108910875
OtLOC111426777
OtLOC111426716
OtLOC111426766
OtLOC111423866
c
CsLOC6047053

Figure S1. Chromosomal locations and duplications modes of identified GH1s in Hymenoptera, Coleoptera, Diptera. Gene names are presented with two-letter species abbreviations and gene ids; red, blue, black, and yellow gene names represent tandem duplications, proximal duplications, dispersed duplications, and segmental duplications, respectively. Different color of karyotype indicates different species and red lines link the GH1 genes from collinear blocks. a) Hymenoptera: Ac, *Atta colombica*; Am, *Apis mellifera*; Bt, *Bombus terrestris*; Nv, *Nasonia vitripennis*; Pd, *Polistes dominula*; b) Coleoptera: Ot, *Onthophagus taurus*;An, *Anoplophora glabripennis*; Tc, *Tribolium castaneum*; Al, *Agrilus planipennis*; c) Diptera: Aa, *Aedes aegypti*; Cq, *Culex quinquefasciatus*; Dm, *Drosophila melanogaster*; Ag, *Anopheles gambiae*.

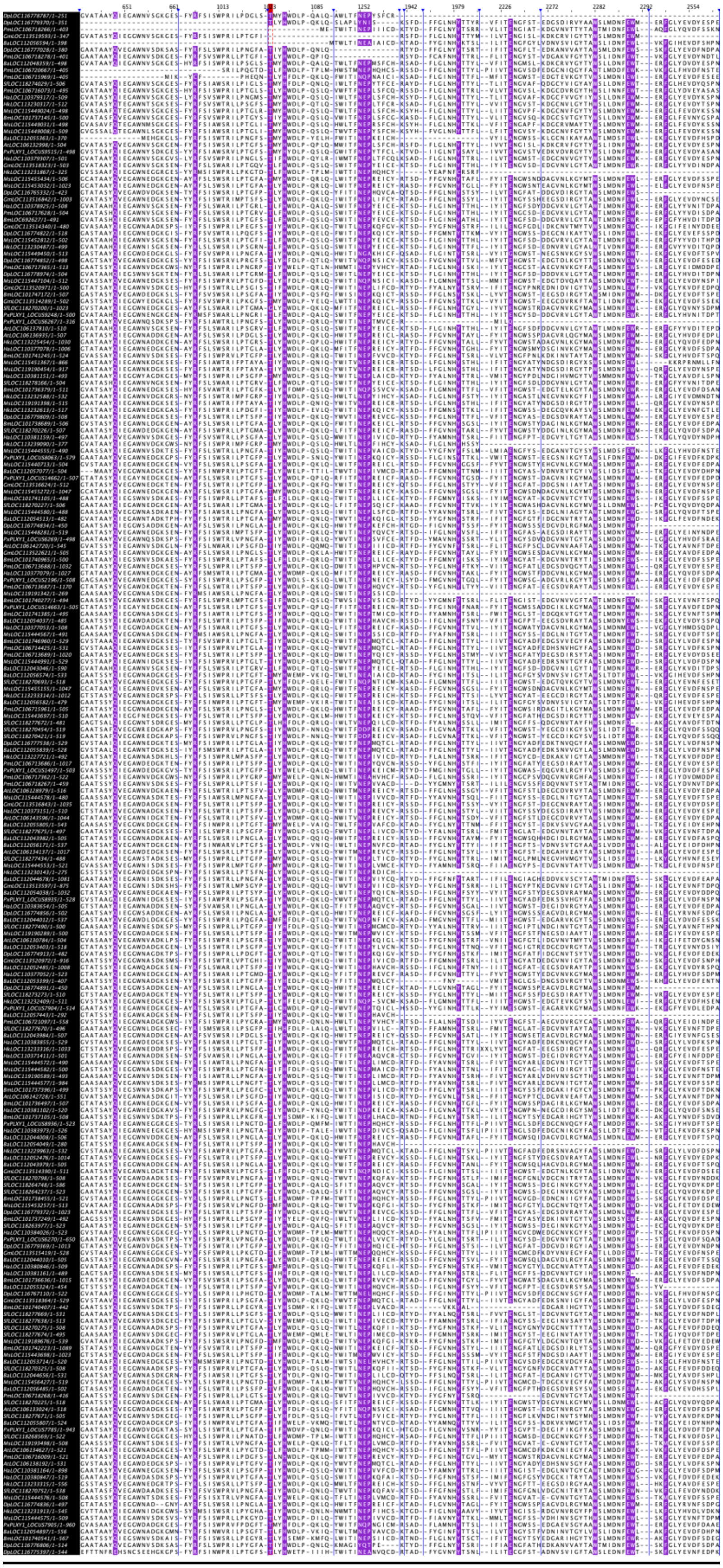
651
661
1013
1085
1252
1942
1979
2226
2272
2282
2554

Figure S2. A part of the alignment of lepidopteran GH1s. Functionally important sites, including binding sites, catalytic sites, and residues next to active centers, were labeled in purple.
